# Interferon- **α** 2b reduces phosphorylation and activity of MEK and ERK through a *Ras* / *Raf* -independent mechanism

**DOI:** 10.1054/bjoc.2000.1263

**Published:** 2000-07-24

**Authors:** F Romerio, A Riva, D Zella

**Affiliations:** Institute of Human Virology, University of Maryland Biotechnology Institute (UMBI), 725 West Lombard Street, Baltimore, Maryland 21201, USA

**Keywords:** IFN-α, cellular proliferation, MEK/ERK pathway

## Abstract

Interferon (IFN)-α affects the growth, differentiation and function of various cell types by transducing regulatory signals through the Janus tyrosine kinase/signal transducers of activation and transcription (Jak/STAT) pathway. The signalling pathways employing the mitogen-activated ERK-activating kinase (MEK) and the extracellular-regulated kinase (ERK) are critical in growth factors signalling. Engagement of the receptors, and subsequent stimulation of *Ras* and *Raf*, initiates a phosphorylative cascade leading to activation of several proteins among which MEK and ERK play a central role in routing signals critical in controlling cell development, activation and proliferation. We demonstrate here that 24–48 h following treatment of transformed T- and monocytoid cell lines with recombinant human IFN-α2b both the phosphorylation and activity of MEK1 and its substrates ERK1/2 were reduced. In contrast, the activities of the upstream molecules *Ras* and *Raf* -1 were not affected. No effect on MEK/ERK activity was observed upon short-term exposure (1–30 min) to IFN. The anti-proliferative effect of IFN-α was increased by the addition in the culture medium of a specific inhibitor of MEK, namely PD98059. In conclusion, our results indicate that IFN-α regulates the activity of the MEK/ERK pathway and consequently modulates cellular proliferation through a *Ras* / *Raf* -independent mechanism. Targeting the MEK/ERK pathway may strengthen the IFN-mediated anti-cancer effect. © 2000 Cancer Research Campaign


*British Journal of Cancer* (2000) **83**(4), 532–538
© 2000 Cancer Research Campaign
doi: 10.1054/ bjoc.2000.1263, available online at http://www.idealibrary.com on IDEAL®

### Interferon-α2b reduces phosphorylation and activity of MEK and ERK through a *Ras*/*Raf*-independent mechanism

**F Romerio[1], A Riva[2] and D Zella[1]**

[1]Institute of Human Virology, University of Maryland Biotechnology Institute (UMBI), 725 West Lombard Street, Baltimore, Maryland 21201, USA;
[2]Istituto di Malattie Infettive, Ospedale Luigi Sacco, Universitá di Milano, 20100 Milano, Italy

**Summary** Interferon (IFN)-α affects the growth, differentiation and function of various cell types by transducing regulatory signals through the Janus tyrosine kinase/signal transducers of activation and transcription (Jak/STAT) pathway. The signalling pathways employing the mitogen-activated ERK-activating kinase (MEK) and the extracellular-regulated kinase (ERK) are critical in growth factors signalling. Engagement of the receptors, and subsequent stimulation of *Ras* and *Raf*, initiates a phosphorylative cascade leading to activation of several proteins among which MEK and ERK play a central role in routing signals critical in controlling cell development, activation and proliferation. We demonstrate here that 24–48 h following treatment of transformed T- and monocytoid cell lines with recombinant human IFN-α2b both the phosphorylation and activity of MEK1 and its substrates ERK1/2 were reduced. In contrast, the activities of the upstream molecules *Ras* and *Raf*-1 were not affected. No effect on MEK/ERK activity was observed upon short-term exposure (1–30 min) to IFN. The anti-proliferative effect of IFN-α was increased by the addition in the culture medium of a specific inhibitor of MEK, namely PD98059. In conclusion, our results indicate that IFN-α regulates the activity of the MEK/ERK pathway and consequently modulates cellular proliferation through a *Ras*/*Raf*-independent mechanism. Targeting the MEK/ERK pathway may strengthen the IFN-mediated anti-cancer effect. © 2000 Cancer Research Campaign

**Keywords**: IFN-α; cellular proliferation; MEK/ERK pathway

The Jak/STAT pathway is used by IFN-α and other cytokines to transduce regulatory signals inside the cell (Darnell, 1997). In this pathway, receptor-associated, ligand-activated kinases phosphorylate STAT proteins on tyrosine residues. Once modified and activated, dimerized STATs translocate into the nucleus to initiate trascriptional regulation (Darnell, 1997), affecting the growth, differentiation and function of various cell types (for review *see* Herberman, 1997). The signalling pathways employing MEK and ERK are critical in growth factors signalling (Pages et al, 1993; Cowley et al, 1994; Seger and Krebs, 1995; Pumiglia and Decker, 1997; Madhani and Fink, 1998). MEK is activated by upstream molecules, among which *Ras* and *Raf* play a fundamental role.

Due to the critical involvement of the MEK/ERK pathway in the events controlling cellular proliferation, and to the well documented antiproliferative effect of IFN-α (Pfeffer et al, 1998), we tested the effects of IFN-α on the MEK/ERK pathway, in an attempt to provide a molecular basis for the anti-proliferative activity of this multifunctional cytokine. We demonstrate here that recombinant human IFN-α2b reduces the phosphorylation and activity of both MEK1 and ERK1/2 through a *Ras*/*Raf*-independent mechanism.

*Received 16 December 1999*
*Revised 3 April 2000*
*Accepted 6 April 2000*

*Correspondence to:* D Zella

### MATERIALS AND METHODS

### Transformed cell lines and reagents

The CD4+ lymphoblastoid cell lines Jurkat, SupT1, H9 and CEM, and the monocytoid cell line U937 (all from ATCC, Manassas, VA, USA) were maintained in complete RPMI medium supplemented with 10% fetal bovine serum (GIBCO/BRL, Gaithersburg, MD, USA). Treatment with IFN-α was performed at the indicated concentrations by culturing the cells at $3–5 \times 10^5$ cells $ml^{-1}$ in complete RPMI medium. Recombinant human IFN-α2b was from Biosidus (Buenos Aires, Argentina). The protein was > 98% pure as assessed by gel electrophoresis. IFN-α antiviral activity was assessed in culture by the standard biological test by using MDBK cells and vescicular stomatitis virus, as previously described (Rubinstein et al, 1981). The activity was in the range of $2–3 \times 10^8$ U $mg^{-1}$. Anti-IFN-α polyclonal antibodies were from Biosource International, Camarillo, CA, USA. The MEK1 inhibitor PD98059 was purchased from New England Biolabs (Beverly, MA, USA).

### Western blot

Aliquots of cells were lysed in a buffer containing 20 mmol $l^{-1}$ Tris-Cl (pH 7.5), 150 mmol $l^{-1}$ NaCl, 1 mmol $l^{-1}$ EDTA, 1 mmol $l^{-1}$ EGTA, 1% Triton X-100, 2.5 mmol $l^{-1}$ sodium pyrophosphate, 1 mmol $l^{-1}$ β-glycerolphosphate, 1 mmol $l^{-1}$ $Na_3VO_4$, 1 μg $ml^{-1}$ leupeptin, 1 mmol $l^{-1}$ PMSF. The lysate was incubated at 4°C for 15 min with moderate shaking. Cell debris were pelleted by centrifugation, and the supernatant collected and frozen. 25 μg of whole-cell lysate were run on a 12% SDS-PAGE in Tris-glycine buffer. The gels were transferred to a PVDF membrane using a SemiDry blotting apparatus (Pharmacia, Piscataway, NJ, USA) in

25 mmol $l^{-1}$ Tris, 192 mmol $l^{-1}$ glycine, 20% methanol. After blocking in Blotto-Tween (10 mmol $l^{-1}$ Tris-Cl (pH 7.5), 0.9% NaCl, 0.1% Tween-20, 5% Nonfat Dry Milk), the membranes were first probed with antibodies detecting phospho-proteins (according to the manufacturer's instructions) and developed using the ECL Plus Kit from Amersham (Arlington Heights, IL, USA). Subsequently, the blots were stripped in 0.1 mol $l^{-1}$ glycine (pH 2.9), blocked in Blotto-Tween and re-probed with antibodies detecting total protein levels. Anti-ERK1/2, anti-MEK1, anti-*Raf*-1, anti-*p38* and anti-β-actin antibodies were from Santa Cruz Biotechnology (Santa Cruz, CA, USA). Anti-phospho-ERK1/2, anti-phospho-MEK1/2 and anti-phospho-*p38* antibodies were from New England Biolabs (Beverly, MA, USA). The effect of IFN-α on the rate of re-phosphorylation of ERK1/2 in Jurkat cells was investigated by either of two protocols. In the first procedure, the cells were cultured for 24–48 h in medium supplemented with 0.5% serum, and then re-stimulated with 10% serum (for 0–30 min) in the presence or absence of IFN-α. In the second procedure, the cells were cultured for 24–48 h in 0.5% serum with or without IFN-α, and subsequently re-stimulated with 10% serum alone (for 0–30 min). Whole-cell lysates were prepared as described above, immediately after collection of each aliquot.

### Immunoprecipitation and kinase assay for MEK and ERK activity

The ERK1/2 and MEK1/2 kinase assays were performed using kits available from New England Biolabs, following the manufacturer's instructions. Quantification of all blots was performed by densitometric analysis using the Molecular Analyst® software (version 2.1) from BioRad (Hercules, CA, USA).

### Assessment of in vivo $p21^{ras}$ activity

The assay to assess the GTPase activity in IFN-α-treated vs untreated cells was performed as previously described (Burgering et al, 1991) with few minor modifications. Cells were grown for 24–48 h with or without 30 ng $ml^{-1}$ IFN-α. Subsequently, the cells were labelled with 40 μCi $ml^{-1}$ carrier-free $[^{32}P]H_3PO_4$ (ICN, Costa Mesa, CA, USA) for 3 h at 37°C in RPMI 1640 without sodium phosphate. Control aliquots were removed, and the remaining cells were stimulated by cross-linking with anti-CD3/CD4 antibodies as described (Kirk and Miller, 1998). Cells were washed in ice-cold PBS to remove the unincorporated radioactive phosphate, and then lysed in 500 μl of ice-cold lysis buffer containing 50 mmol $l^{-1}$ Tris-Cl (pH 7.5), 1% Triton X-114, 150 mmol $l^{-1}$ NaCl, 5 mmol $l^{-1}$ $MgCl_2$, 10 μg $ml^{-1}$ leupeptin, 10 μg $ml^{-1}$ aprotinin, 1 mmol $l^{-1}$ PMSF, 100 μmol $l^{-1}$ GTP, 100 μmol $l^{-1}$ GDP, 1 mmol $l^{-1}$ $NaPO_4$ (pH 7.5). After 15 min on ice, nuclei were removed by centrifugation at 4°C. In order to recover the membrane-associated (active) fraction of *Ras*, the lysate was incubated at 37°C for 3 min followed by a brief centrifugation at room temperature. The upper aqueous phase was removed and the lower Triton phase diluted 10-fold with lysis buffer without Triton X-114. An equal number of cpm were incubated in a 500-μl reaction with 20 μl of agarose-conjugated anti-*p21* antibody (clone Y13-259, Santa Cruz Biotechnology, Santa Cruz, CA, USA) for 3 h at 4°C with end-over-end agitation. The immunoprecipitates were washed eight times with 1 ml of wash buffer containing 50 mmol $l^{-1}$ Tris-Cl (pH 7.5), 500 mmol $l^{-1}$ NaCl, 5 mmol $l^{-1}$ $MgCl_2$, 0.1% Triton X-100, 0.005% SDS. *Ras*-bound nucleotides were eluted in 20 μl of elution buffer (40 mmol $l^{-1}$ EDTA, 40 mmol $l^{-1}$ Tris-Cl pH 7.5, 4% SDS, 1 mmol $l^{-1}$ GTP, 1 mmol $l^{-1}$ GDP) by incubation at 68°C for 20 min. An equal number of cpm were spotted on PEI-Cellulose plates (JT Baker, Phillipsburg, NJ, USA) and developed for 1 h in 1.2 mol $l^{-1}$ ammonium formate, 0.8 mol $l^{-1}$ HCl. The plates were air-dried and exposed to a Storm PhosphorImager for quantification of the GTP and GDP spots.

### In vitro Raf activity assay

*Raf*-1 was immunoprecipitated from whole cell lysates using specific antibodies (Santa Cruz). The immuno-complexes were washed three times in lysis buffer and then in kinase assay buffer (25 mmol $l^{-1}$ Tris-Cl (pH 7.5), 5 mmol $l^{-1}$ β-glycerolphosphate, 2 mmol $l^{-1}$ DTT, 0.1 mmol $l^{-1}$ $Na_3VO_4$ and 10 mmol $l^{-1}$ $MgCl_2$). Kinase reactions were performed by incubating the immuno-complexes at 30°C for 30 min in the presence of 200 μmol $l^{-1}$ ATP and 2 μg of the specific substrate (MEK 1, from Santa Cruz) in kinase buffer. Reactions were stopped with SDS-PAGE loading buffer and run on a 12% SDS-PAGE. The gels were transferred to PVDF and probed with an anti-phospho-MEK1/2 antibody. Blots were subsequently stripped and reprobed with an anti-*Raf*-1 antibody to confirm the presence of equal amounts of *Raf*-1 in all lanes.

### RESULTS

### IFN-α treatment reduces proliferative capacity of transformed cell lines

The anti-proliferative effect of IFN-α was first determined in several lymphocytoid transformed CD4+ cell lines, namely Jurkat, U937, SupT1, H9 and CEM. As reported in Figure 1A, all cell lines were sensitive to treatment with IFN-α, displaying a reduction in proliferation ranging from 10–25% at day 2 to 35–40% at day 6 of culture. The dose- and time-dependent anti-proliferative effect was also assessed. Cultures of Jurkat cells were maintained for 6 days in the presence of IFN-α at a concentration range of 0.1–100 ng $ml^{-1}$ (Figure 1B). This experiment shows that by day 6 concentrations of IFN-α as low as 0.5 ng $ml^{-1}$ (corresponding to 150 IU $ml^{-1}$) had a remarkable inhibitory effect on the proliferation of Jurkat cells. Since a concentration of IFN-α of 10–50 ng $ml^{-1}$ was sufficient to reduce cell proliferation by about 30% at day 4 and about 50% at day 6, all subsequent experiments were carried out by culturing the cells in the presence of 30 ng $ml^{-1}$ of IFN-α.

### Treatment with IFN-α reduces the levels of phosphorylation and activity of MEK1 and ERK1/2 in Jurkat and U937

Once activated by autophosphorylation or upstream kinases (Gomez and Cohen, 1991; Dent et al, 1992; Howe et al, 1992; Kyriakis et al, 1992; Ahn et al, 1993; Itoh et al, 1993; Matsuda et al, 1993; Posada et al, 1993; Gardner et al, 1994; Haystead et al, 1994; Papin et al, 1998), MEK activity is the main upstream mechanism leading to phosphorylation of both tyrosine and serine/threonine residues and subsequent activation of ERK (Seger and Krebs, 1995). This, in turn, results in the regulation of a large number of proteins both in the cytoplasm and, following ERK translocation, in the nucleus (Khokhlatchev et al, 1998).

© 2000 Cancer Research Campaign

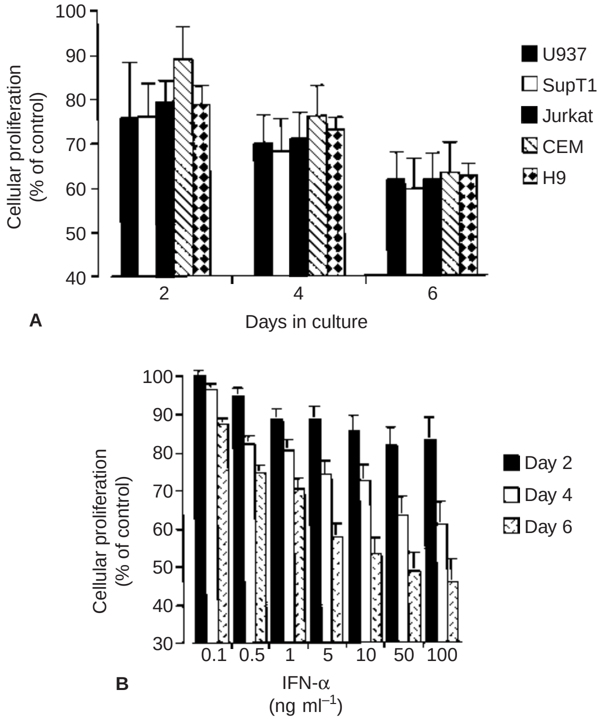

**Figure 1** (**A**) Effect of IFN-α on the proliferation of several CD4+ cell lines. Cells were grown for 6 days in complete RPMI medium containing 30 ng $ml^{-1}$ IFN-α. Every 2 days, samples of the cultures were taken, and cell count and viability (as determined by triypan blue exclusion) were determined. The data shown are representative of the results obtained in four independent experiments carried out in duplicate with Jurkat, SupT1, H9, CEM and U937. Mean and standard deviation are shown. (**B**) Dose- and time-dependent anti-proliferative effect of IFN-α on Jurkat cells. Cells were cultured for 6 days in the presence of IFN-α (0.1–100 ng $ml^{-1}$), and samples of the cultures were taken at days 2, 4 and 6. Cell count and viability were determined by trypan blue staining. Results are representative of four independent experiments carried out with duplicate samples. Addition of polyclonal antibodies against IFN-α reversed its anti-proliferative effect and during the 6 days of experiment the number of viable cells was constantly above 95% (not shown)

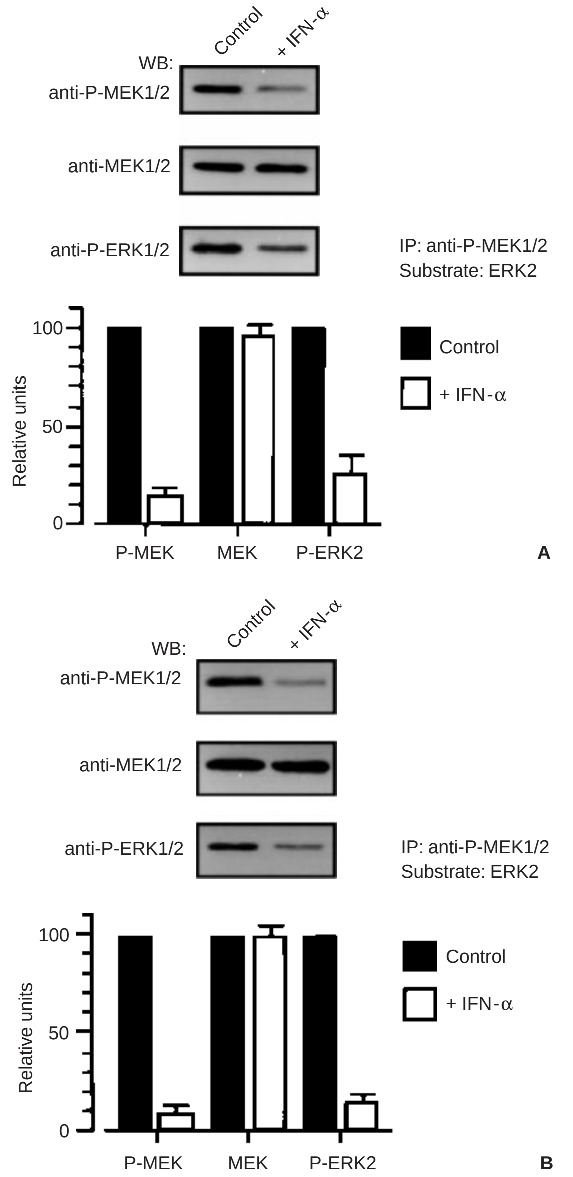

**Figure 2** Treatment with IFN-α specifically reduces phosphorylation and activity of MEK1. Western blot analysis of the phosphorylation, protein levels and kinase activity assay of MEK1 is shown. (**A**) Jurkat and (**B**) U937 cell lines were treated with IFN-α (30 ng $ml^{-1}$) and after 48 h whole-cell extracts were prepared as described in Methods. Equal amounts of total protein were loaded in each lane or used to immunoprecipitate phospho-MEK1, and subsequently analysed for the phosphorylation and activity of MEK1, respectively. Upper panel = amounts of phosphorylated MEK1 (P-MEK1), middle panel = total MEK1, and lower panel = activity of P-MEK1 on its substrate, ERK2. A representative of three independent experiments with each cell line is shown. At the bottom of the figure, a histogram is reported with mean and standard deviation of the relative values of the densitometric analyses from three independent experiments

Due to its importance in the process leading to cellular proliferation, we first determined the effect of IFN-α on MEK phosphorylation and kinase activity. Preliminary experiments carried out in our laboratory have indicated that the use of a Triton X-100-based buffer to prepare whole-cell lysates (see Materials and Methods for details) allows the recovery of greater than 95% of MEK1 and ERK1/2 in the soluble fraction (not shown). In addition, side-by-side experiments have indicated that preparation of whole-cell lysates with a Triton X-100-based buffer or by boiling the cells in SDS-PAGE sample buffer yields identical results (not shown). Since the former procedure is carried out in native conditions and allows the recovery of enzymatically active proteins that can be used in kinase assays, we have employed this method for the preparation of cell lysates throughout this study (except for those used to assess *Ras* activity, see Materials and Methods).

As representative of transformed CD4+ cell lines, we selected a monocytoid cell line (U937) and a lymphocytoid cell line (Jurkat). Upon treatment with IFN-α for 48 h, we observed a drastic reduction in MEK1 phosphorylation at Ser 217 and Ser 221 in both cell lines, as assessed by Western blot with specific antibodies (Figures 2A and 2B, upper panel). This reduced phosphorylation paralleled a reduction in kinase activity, whereas the amount of total protein did not change (Figures 2A and 2B, lower and middle panel, respectively). We determined by densitometric analysis the relative intensity of each band from several independent experiments: the mean and the standard deviation are plotted in the histograms. Subsequently, we determined the status of phosphorylation and the activity of the downstream kinases, ERK1/2. In agreement with the results obtained for MEK1, we observed a drastic reduction in ERK1/2 phosphorylation at Thr 202 and Tyr 204 in both cell lines,

 © 2000 Cancer Research Campaign

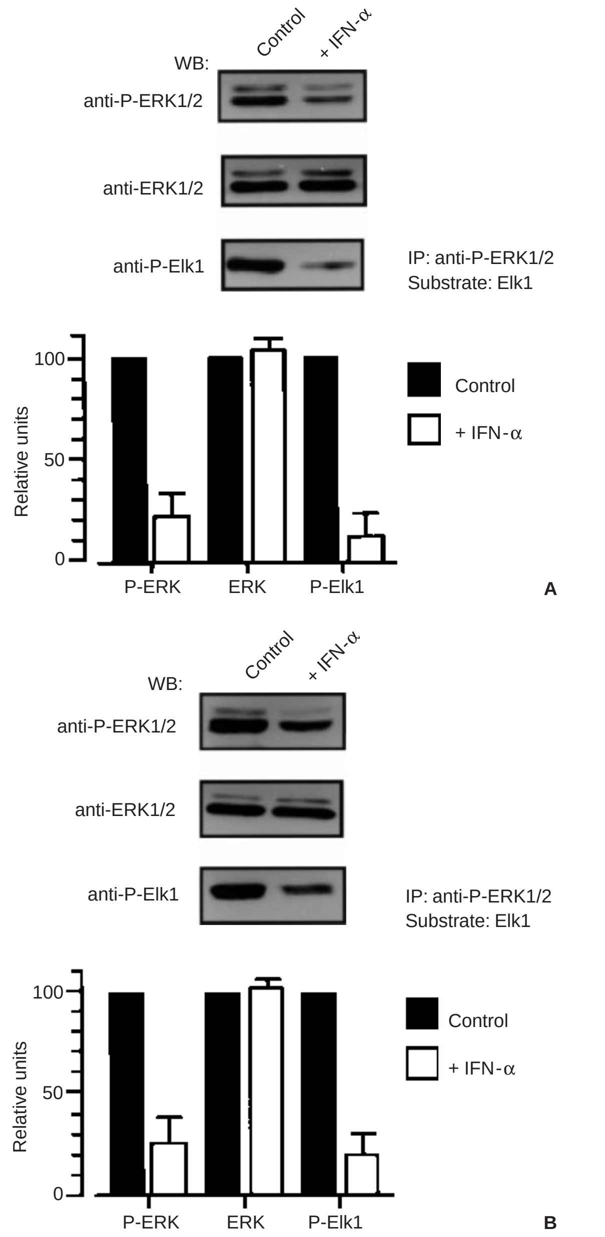

**Figure 3** Treatment with IFN-α specifically reduces phosphorylation and activity of ERK1/2. Western blot analysis of the phosphorylation level, total protein amount and kinase activity assay of ERK1/2 is shown. (**A**) Jurkat and (**B**) U937 cell lines were treated with IFN-α (30 ng $ml^{-1}$) and after 48 h whole-cell extracts were prepared as described in Methods. Equal amounts of total protein were loaded in each lane or used to immunoprecipitate phospho-ERK1/2, and subsequently analysed for the phosphorylation and activity of ERK1/2, respectively. Upper panel = amounts of phosphorylated ERK1/2 (P-ERK1/2), middle panel = total ERK1/2, and lower panel = activity of P-ERK1/2 on its substrate, Elk-1. A representative of three independent experiments with each cell line is shown. At the bottom of the figure, a histogram is reported with mean and standard deviation of the relative values of the densitometric analyses from three independent experiments

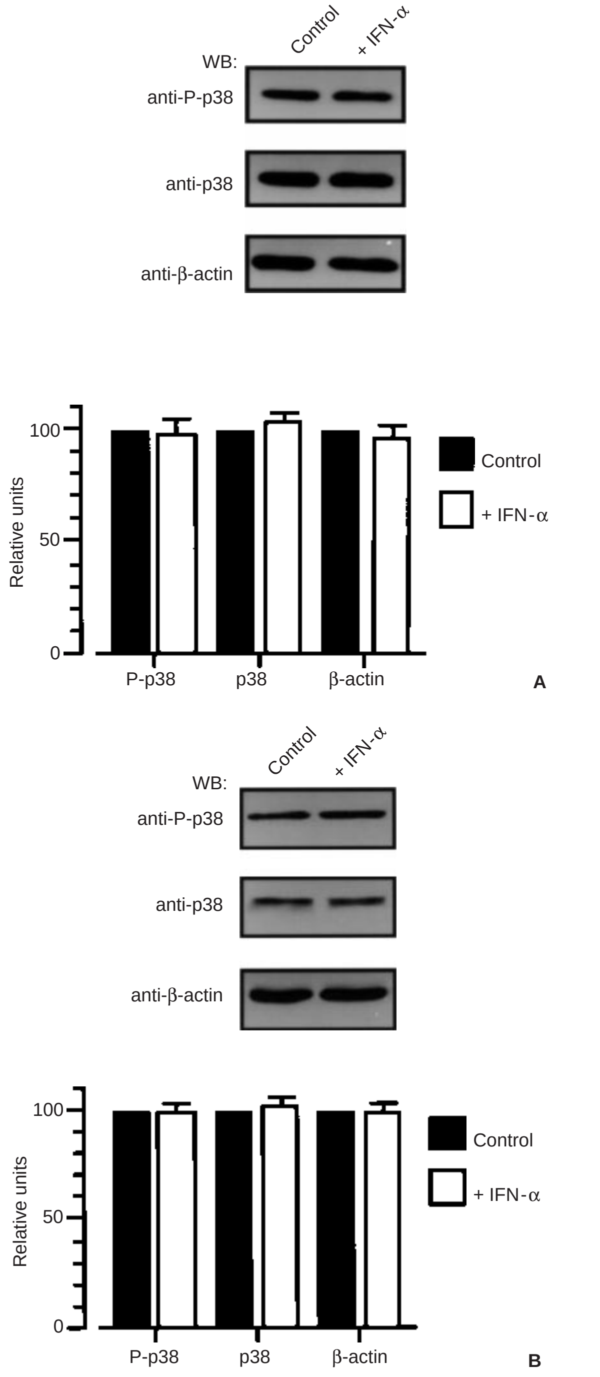

**Figure 4** Treatment with IFN-α does not affect levels and phosphorylation of *p38*. Western blot analysis of the total amount of protein and phosphorylation level of *p38* is shown (Itoh et al, 1993). (**A**) Jurkat and (**B**) U937 cell lines were treated with IFN-α (30 ng $ml^{-1}$) and after 48 h total cellular extracts were prepared as described in Methods. Equal amounts of total protein were loaded in each lane and subsequently analysed for the levels and phosphorylation of *p38*, as described in Methods. Upper panel = amounts of phosphorylated *p38* (P-*p38*), middle panel = total *p38*, and lower panel = β-actin. A representative of three independent experiments with each cell line is shown. At the bottom of the figure a histogram is reported with mean and standard deviation of the relative values of the densitometric analyses from three independent experiments while the total amount of protein was stable (Figure 3A and 3B, upper panel). Consistent with this result, we detected a strong reduction in the kinase activity of ERK1/2 on the substrate Elk1 in both cell lines (Figure 3A and 3B, lower panel). However the protein levels of ERK1/2 remained unchanged (Figure 3A and 3B, middle panel). Phosphorylation of another MAPK implicated in the cell cycle regulation (namely *p38*) (Takenaka et al, 1998) was not affected (Figure 4A and 4B, upper panel), thus suggesting a specific action of IFN-α on the MEK/ERK pathway.

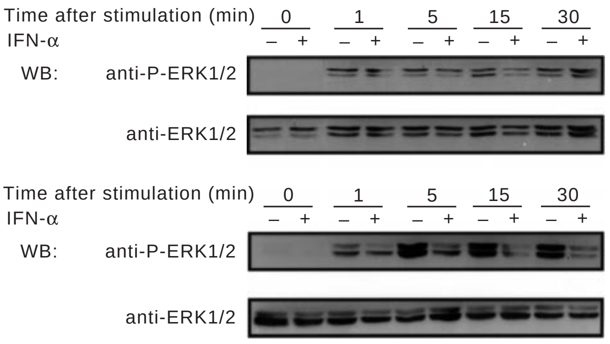

**Figure 5** Short-term treatment of Jurkat cells with IFN-α does not affect the rate of activation of ERK1/2 in response to serum. In the upper panel, cells were grown in low serum for 24–48 h and subsequently re-stimulated with serum either in the presence or the absence of IFN-α. In the lower panel, cells were first cultured for 24–48 h in the presence or absence of IFN-α and then re-stimulated with serum alone. As shown, the presence of IFN-α during re-stimulation with serum does not influence the rate of re-phosphorylation of ERK1/2, while the presence of IFN-α during serum deprivation strongly affects the kinetic of re-activation of ERK1/2. The results shown are representative of three independent experiments

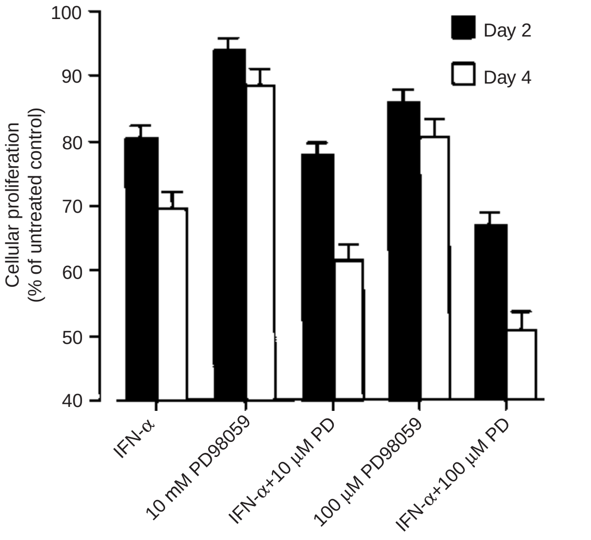

**Figure 6** Additive effect of IFN-α and PD98059 in reducing cellular proliferation. Jurkat cells were grown in complete RPMI medium as described in Materials and Methods, supplemented with 30 ng $ml^{-1}$ of IFN-α and 10 or 100 µmol $l^{-1}$ of PD98059, or a combination of the two molecules. At days 2 and 4 cell counts and viability were determined by trypan blue exclusion. The data shown represent results obtained in four independent experiments carried out with duplicate samples. Mean and standard deviation are indicated

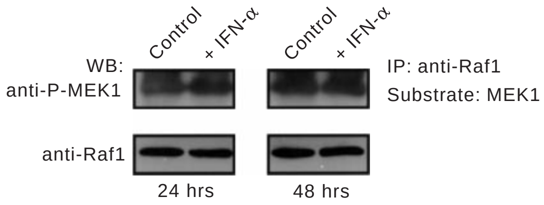

**Figure 7** Treatment with IFN-α does not affect the kinase activity of *Raf*-1. Jurkat cells were grown for 24 and 48 h in complete RPMI medium containing 30 ng $ml^{-1}$ IFN-α. Equal amounts of whole cell lysate were used to immunoprecipitate *Raf* from IFN-α-treated and control cells, and subsequently to determine the kinase activity of *Raf* using MEK1 as a substrate (upper panel). The lower panel shows that equal amounts of *Raf* were present in the immunocomplexes from treated and untreated cells. The results shown are representative of five independent experiments

### Inhibition of the MEK/ERK pathway is a long-term effect of IFN-α treatment

As other groups have shown that short-term treatment with IFN-α can activate the MEK/ERK pathway (Arora et al, 1999; Lund et al, 1999), we also investigated the possibility that brief exposures to IFN-α may affect the activity of MEK/ERK. When we treated Jurkat cells with IFN-α for short periods of time (1–30 min) we were unable to observe any increase in the phosphorylation of ERK1/2 (not shown). However, we observed that the baseline level of phosphorylation of ERK1/2 in normally growing Jurkat cells was very high and possibly already reached its maximum, raising the possibility that this could have masked the effect of IFN-α, thereby preventing us from appreciating any increase in the phosphorylation of ERK1/2. Therefore we designed an experiment that could allow us to observe any stimulatory effect of IFN-α on the phosphorylation of ERK1/2. First we cultured Jurkat cells in low-serum medium for 24–48 h, which markedly diminished the phosphorylation of ERK1/2, and then we tested whether short-term exposures to IFN-α could increase the rate of re-phosphorylation of ERK1/2 upon addition of serum to the culture medium. As shown in the upper panel of Figure 5, IFN-α has no influence on the rate of activation of ERK1/2 after the addition of serum. In addition, treatment of serum-deprived Jurkat cells with IFN-α alone had no effect on the re-phosphorylation of ERK1/2 (not shown). However, Jurkat cells cultured for 24–48 h in low-serum medium supplemented with IFN-α showed a much slower kinetic of re-phosphorylation of ERK1/2 upon addition of serum, as compared to cells cultured in low-serum medium alone.

Finally, we assessed whether in our in vitro model the reduction in the activity of MEK/ERK is a molecular phenomenon consequent to – as opposed to the cause of – the diminished cellular proliferation observed after treatment with IFN-α. For this reason, we sought to uncouple cellular proliferation from MEK/ERK pathway activity. First we attempted to diminish the rate of cellular proliferation while leaving the activity of MEK/ERK unaffected, by culturing the cells in progressively lower concentrations of serum (not shown). Second, we attempted to block the activity of MEK/ERK while leaving the rate of cellular proliferation unchanged, by adding PD98059, a specific inhibitor of MEK1/2, to the culture medium. We were unsuccessful in our attempt to dissociate the activity of the MEK/ERK pathway from cellular proliferation. In fact, cells cultured in the presence of both IFN-α and PD98059 showed a dose-dependent additive effect in reducing cellular proliferation, as compared to cells treated with IFN-α alone (Figure 6). These data indicate that in our in vitro system cellular proliferation requires an active and functional MEK/ERK pathway and that IFN-α reduces cellular proliferation by interfering with this pathway.

### Treatment with IFN-α does not affect the activities of *Ras* and *Raf*

The major mechanism leading to MEK activation requires the upstream kinase, *Raf*-1, and GTPase, *Ras* (Dent et al, 1992; Howe et al, 1992; Kyriakis et al, 1992). To determine whether IFN-α

 © 2000 Cancer Research Campaign

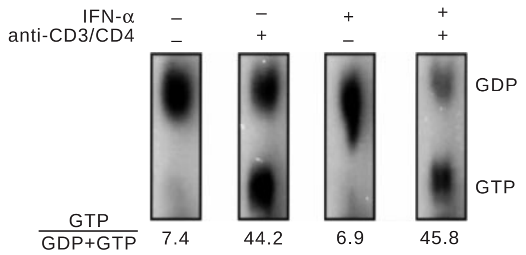

**Figure 8** Treatment with IFN-α does not alter the GTPase activity of *Ras*. Jurkat cells were grown for 48 h in complete RPMI medium in the presence or absence of 30 ng $ml^{-1}$ IFN-α, and subsequently metabolically labelled with radioactive orthophosphoric acid. Whole-cell lysates were prepared before and after stimulation of the IFN-α-treated and untreated cells with cross-linked anti-CD3/CD4 antibodies. Equal amounts of whole-cell lysate were used to immunoprecipitate *Ras*. Radioactive *Ras*-associated nucleotides were eluted and separated by thin-layer chromatography. As shown, treatment with IFN-α does not affect the ability of Jurkat cells to transiently activate the GTPase activity of *Ras* in response to the stimulatory signal. The results shown are representative of five independent experiments reduces MEK/ERK activity through a *Ras*/*Raf*-dependent mechanism, we assessed the activity of *Raf*-1 and *Ras* recovered from IFN-treated Jurkat cells. As shown in Figure 7 (upper panel), kinase activity of *Raf*-1 recovered from Jurkat cells treated with IFN-α for 24 and 48 h was not altered as compared to untreated controls. The lower panel (Figure 7) shows that identical amounts of *Raf*-1 protein were immunoprecipitated from all samples.

Consistent with the results obtained for *Raf*, we observed similar amounts of GTP-bound, active *Ras* immunoprecipitated from lysates of IFN-treated or control cells after stimulation with cross-linked anti-CD3/CD4 antibodies (Figure 8). Similar results have been obtained using PMA (100 ng $ml^{-1}$) to induce transient activation of *Ras* (not shown). Western blot assays confirmed that treatment with IFN-α does not alter the expression of *Ras* (not shown). Overall, these results point toward a mechanism responsible for the IFN-mediated reduction of MEK/ERK activity, which bypasses the upstream molecules, *Ras* and *Raf*-1.

### DISCUSSION

Here we have shown that treatment with IFN-α reduces the phosphorylation of MEK1 and ERK1/2 in transformed cell lines. The diminished phosphorylation was observed after 24–48 h and was paralleled by reduced enzymatic activity as assessed by phosphorylation assays of specific substrates (namely ERK2 and E1k-1 for MEK1 and ERK1/2, respectively). In addition, since Western blot analysis demonstrated the same amount of MEK and ERK proteins, the results cannot be attributed to a direct mechanism whereby IFN-α reduces the expression of MEK1 and ERK1/2. Moreover, the reduction in the activity of the MEK/ERK pathway was associated with a reduction of proliferative capacity of transformed cell lines. Given the fundamental importance of this pathway in regulating cell proliferation, this reduction in the activity of MEK/ERK may constitute a molecular mechanism for the anti-proliferative activity of IFN-α. Finally, the decrease MEK/ERK activity was not associated with reduction of *Ras*/*Raf* function.

Although the Jak/STAT pathway is clearly important in the establishment of the IFN-mediated effects, a number of recent studies suggest that additional signalling pathways may also be important for IFN-dependent biological response. To this regard, type I IFNs have been shown to stimulate *Raf* and ERK activation in a Jak-dependent, but *Ras*-independent manner (Stancato et al, 1997; Sakatsume al, 1998). The cross-talk between the Jak/STAT and MEK/ERK pathway is further demonstrated by observation that stimulation with IFN-β can result in activation of ERK and its direct association with STAT1, as revealed by co-immunoprecipitation studies (David et al, 1995). In other reports IFN-α has been shown to directly activate MEK/ERK (Arora et al, 1999; Lund et al, 1999) and phosphatidylinositol 3-kinase (PI-3K) (Uddin et al, 1997). In contrast, treatment of cells with the PI-3K inhibitor wortmannin appears to inhibit type I IFN-regulated ERK activation (Uddin et al, 1997). It is important to note that all these experiments analysed events occurring very early after the stimulation of cells with IFN-α. Certainly, this is a notable contribution to the understanding of all the molecular players involved in the establishment of the IFN-mediated effects. Nonetheless, biochemical and functional characterization of the long-term events occurring upon treatment with IFN-α are also needed to better comprehend the mechanisms of action of this multifunctional cytokine.

A number of experiments investigated the long-term molecular effects of exposure to IFN-α. Examples of such effects include modulation of PKC isotypes, down-regulation of *c-myc* and cyclin-A, and hypophosphorylation of the retinoblastoma gene (Resnitky et al, 1992). In addition, it was demonstrated that transcriptionally active STAT1 is required for the anti-proliferative effect of IFN-α, and this anti-proliferative activity was established several hours after initial treatment of the cells with IFN-α (Bromberg et al, 1996). Proliferation of STAT1-deficient cells was not inhibited by IFN-α (Bromberg et al, 1996; Grimley et al, 1998). Finally, failure of IFN-α to reduce proliferative ability of a cutaneous T-cell lymphoma (CTCL) cell line was correlated to lack of STAT1 expression (Sun et al, 1998). These observations strongly suggest that: i) activation of the Jak/STAT pathway is crucial to establish the anti-proliferative effect of IFN-α; ii) it is likely that events occurring downstream of this pathway, such as effects on the rate of protein synthesis, are relevant for the establishment of IFN-mediated anti-proliferative effect.

Our results show that exposure to IFN-α results in an impairment of the MEK/ERK pathway, which in turn causes a reduction in rate of cellular proliferation. This conclusion is supported by a recent publication showing that a blockade in the MAPK pathway by a selective inhibitor of MEK prevents the growth of colon carcinomas in mice, and further demonstrating that cellular proliferation strongly requires an active MEK/ERK pathway (Sebolt-Leopold et al, 1999).

Several hypotheses can account for the indirect (long-term) effect of the Jak/STAT pathway on the MEK/ERK signalling cascade. A specific phosphatase – such as PP2A (Anderson et al, 1990) – may be induced by IFN-α, thus resulting in the inactivation of MEK or ERK. Another possibility is that IFN-α may regulate the expression of docking proteins (Pawson and Scott, 1997; Schaeffer et al, 1998; Whitmarsh and Davis, 1998), eventually affecting the interaction between MEK and ERK, or between MEK and upstream kinases. Understanding the exact mechanism is important for our comprehension of the proper function of IFN-α and possibly to develop novel targets to strengthen the IFN-mediated anti-cancer effect.

© 2000 Cancer Research Campaign 

### ACKNOWLEDGEMENTS

We thank RC Gallo and D Zagury for helpful comments and suggestions.

### REFERENCES

Ahn NG, Campbell JS, Seger R, Jensenn AM, Graves LM and Krebs EG (1993) Metabolic labeling of mitogen-activated protein kinase kinase in A431 cells demonstrates phosphorylation on serine and threonine residues. *Proc Natl Acad Sci USA* **90**: 5143–5147

Anderson NG, Maller JL, Tonks NK and Sturgill TW (1990) Requirement for integration of signals from two distinct phosphorylation pathways for activation of MAP kinase. *Nature* **143**: 651–653

Arora T, Floyd-Smith G, Espy MJ and Jelinek DF (1999) Dissociation between IFN-alpha-induced anti-viral and growth signaling pathways. *J Immunol* **162**: 3289–3297

Bromberg JF, Horvath CM, Wen Z, Schreiber RD and Darnell JE Jr (1996) Transcriptionally active Stat1 is required for the antiproliferative effects of both interferon alpha and interferon gamma. *Proc Natl Acad Sci USA* **93**: 7673–7678

Burgering BM, Medema RH, Maassen JA, van de Wetering ML, van der Eb AJ, McCormick F. and Bos JL (1991) Insulin stimulation of gene expression mediated by *p21ras* activation. *EMBO J* **10**: 1103–1109

Cowley S, Paterson H, Kemp P and Marshall CJ (1994) Activation of MAP kinase kinase is necessary and sufficient for PC12 differentiation and for transformation of NIH 3T3 cells. *Cell* **77**: 841–852

Darnell Jr JE (1997) STATs and gene regulation. *Science* **277**: 1630–1635

David M, Petricoin E 3rd, Benjamin C, Pine R, Weber MJ and Larner AC (1995) Requirement for MAP kinase (ERK2) activity in interferon alpha- and interferon beta-stimulated gene expression through STAT proteins. *Science* **269**: 1721–1723

Dent P, Haser W, Haystead TA, Vincent LA, Roberts TM and Sturgill TW (1992) Activation of mitogen-activated protein kinase kinase by v-Raf in NIH 3T3 cells and in vitro. *Science* **257**: 1404–1407

Gardner AM, Vaillancourt RR, Lange-Carter CA and Johnson GL (1994) MEK-1 phosphorylation by MEK kinase, Raf and mitogen-activated protein kinase: analysis of phosphopeptides and regulation of activity. *Mol Biol Cell* **5**: 193–201

Gomez N and Cohen P (1991) Dissection of the protein kinase cascade by which nerve growth factor activates MAP kinases. *Nature* **353**: 170–173

Grimley PM, Fang H, Rui H, Petricoin EF 3rd, Ray S, Dong F, Fields KH, Hu R, Zoon KC, Audet S and Beeler J (1998) Prolonged STAT1 activation related to the growth arrest of malignant lymphoma cells by interferon-alpha. *Blood* **91**: 3017–3027

Haystead CM, Gregory P, Shirazi A, Fadden P, Mosse C, Dent P and Haystead TA (1994) Insulin activates a novel adipocyte mitogen-activated protein kinase kinase kinase that shows rapid phasic kinetics and is distinct from c-Raf. *J Biol Chem* **269**: 12804–12808

Herberman RB (1997) Effect of α-interferon on immune function. *Semin Oncol* **24** (Suppl 9): S9-78–S9-80

Howe LR, Leevers SJ, Gomez N, Nakielny S, Cohen P and Marshall CJ (1992) Activation of the MAP kinase pathway by the protein kinase Raf. *Cell* **71**: 335–342

Itoh T, Kaibuchi K, Masuda T, Yamamoto T, Matsuura Y, Maeda A, Shimizu K and Takai Y (1993) A protein factor for Ras p21-dependent activation of mitogen-activated protein (MAP) kinase through MAP kinase kinase. *Proc Natl Acad Sci USA* **90**: 975–979

Khokhlatchev AV, Canagarajah B, Wilsbacher J, Robinson M, Atkinson M, Goldsmith E and Cobb MH (1998) Phosphorylation of the MAP kinase ERK2 promotes its homodimerization and nuclear translocation. *Cell* **93**: 605–615

Kirk CJ and Miller RA (1998) Analysis of Raf-1 activation in response to TCR activation and costimulation in murine T-lymphocytes: effect of age. *Cell Immunol* **190**: 33–42

Kyriakis JM, App H, Zhang XF, Banerjee P, Brautigan DL, Rapp UR and Avruch J (1992) Raf-1 activates MAP kinase-kinase. *Nature* **358**: 417–421

Lund TC, Medveczky MM and Medveczky PG (1999) Interferon-alpha induction of STATs1, -3 DNA binding and growth arrest is independent of Lck and active mitogen-activated kinase in T cells. *Cell Immunol* **192**: 133–139

Madhani HD and Fink GR (1998) The riddle of MAP kinase signaling specificity. *Trends Genet* **14**: 151–155

Matsuda S, Gotoh Y and Nishida E (1993) Phosphorylation of Xenopus mitogen-activated protein (MAP) kinase kinase by MAP kinase kinase kinase and MAP kinase. *J Biol Chem* **268**: 3277–3281

Pages G, Lenormand P, L'Allemain G, Chambard JC, Meloche S and Pouyssegur J (1993) Mitogen-activated protein kinases p42mapk and p44mapk are required for fibroblast proliferation. *Proc Natl Acad Sci USA* **90**: 8319–8323

Papin C, Denouel-Galy A, Laugier D, Calothy G and Eychene A (1998) Modulation of kinase activity and oncogenic properties by alternative splicing reveals a novel regulatory mechanism for B-Raf. *J Biol Chem* **273**: 24939–24947

Pawson T and Scott JD (1997) Signaling through scaffold, anchoring, and adaptor proteins. *Science* **278**: 2075–2080

Pfeffer LM, Dinarello CA, Herberman RB, Williams BR, Borden EC, Bordens R, Walter MR, Nagabhushan TL, Trotta PP and Pestka S (1998) Biological properties of recombinant α-interferon: 40th Anniversary of the discovery of Interferons. *Cancer Res* **58**: 2489–2499

Posada J, Yew N, Ahn NG, Vande Woude GF and Cooper JA (1993) Mos stimulates MAP kinase in Xenopus oocytes and activates a MAP kinase kinase in vitro. *Mol Cell Biol* **13**: 2546–2553

Pumiglia KM and Decker SJ (1997) Cell cycle arrest mediated by the MEK/mitogen-activated protein kinase pathway. *Proc Natl Acad Sci USA* **94**: 448–452

Resnitzky D, Tiefenbrun N, Berissi H and Kimchi A (1992) Interferons and interleukin 6 suppress phosphorylation of the retinoblastoma protein growth-sensitive hematopoietic cells. *Proc Natl Acad Sci USA* **89**: 402–406

Rubinstein S, Familletti PC and Pestka S (1981) Convenient assay for interferons. *J Virol* **37**: 755–758

Sakatsume M, Stancato LF, David M, Silvennoinen O, Saharinen P, Pierce J, Larner AC and Finbloom DS (1998) Interferon gamma activation of Raf-1 is Jak1-dependent and p21 ras-independent. *J Biol Chem* **273**: 3021–3026

Schaeffer HJ, Catling AD, Eblen ST, Collier LS, Krauss A and Weber MJ (1998) MP1: a MEK binding partner that enhances enzymatic activation of the MAP kinase cascade. *Science* **281**: 1668–1671

Sebolt-Leopold JS, Dudley DT, Herrera R, Van Becelaere K, Wiland A, Gowan RC, Tecle H, Barret SD, Bridges A, Przybranowski, Leopold WR and Saltiel AR (1999) Blockade of the MAP kinase pathway suppresses growth of colon tumours in vivo. *Nat Med* **5**: 810–816

Seger R and Krebs EG (1995) The MAPK signaling cascade. *FASEB J* **9**: 726–735

Stancato LF, Sakatsume M, David M, Dent P, Dong F, Petricoin EF, Krolewski JJ, Silvennoinen O, Saharinen P, Pierce J, Marshall CJ, Sturgill T, Finbloom DS and Larner AC (1997) Beta interferon and oncostatin M activate Raf-1 and mitogen-activated protein kinase through a JAK1-dependent pathway. *Mol Cell Biol* **17**: 3833–3840

Sun WH, Pabon C, Alsayed Y, Huang PP, Jandeska S, Uddin S, Platanias LC and Rosen ST (1998) Interferon-alpha resistance in a cutaneous T-cell lymphoma cell line is associated with lack of STAT1 expression *Blood* **91**: 570–576

Takenaka K, Moriguchi T and Nishida E (1998) Activation of the protein kinase p38 in the spindle assembly checkpoint and mitotic arrest. *Science* **280**: 599–602

Uddin S, Fish EN, Sher DA, Gardziola C, White MF and Platanias LC (1997) Activation of the phosphatidylinositol 3-kinase serine kinase by IFN-alpha. *J Immunol* **158**: 2390–2397

Whitmarsh AJ and Davis RJ (1998) Structural organization of MAP-kinase signaling modules by scaffold proteins in yeast and mammals. *Trends Biochem Sci* **23**: 481–485

© 2000 Cancer Research Campaign
